# Facile fabrication of high-efficiency near-infrared absorption film with tungsten bronze nanoparticle dense layer

**DOI:** 10.1186/1556-276X-9-294

**Published:** 2014-06-11

**Authors:** Seong Yun Lee, Jae Young Kim, Jun Young Lee, Ho Jun Song, Sangkug Lee, Kyung Ho Choi, Gyojic Shin

**Affiliations:** 1 IT Convergence Material R&D Group, Korea Institute of Industrial Technology, Cheonan 331-825, Republic of Korea; 2 Department of Chemical Engineering, Sungkyunkwan University, Suwon 440-746, Republic of Korea

**Keywords:** Nanodistance, Nanoparticles, Double layer, Near-infrared absorption, Surface plasmon resonance, Tungsten bronzes

## Abstract

An excellent transparent film with effective absorption property in near-infrared (NIR) region based on cesium-doped tungsten oxide nanoparticles was fabricated using a facile double layer coating method via the theoretical considerations. The optical performance was evaluated; the double layer-coated film exhibited 10% transmittance at 1,000 nm in the NIR region and over 80% transmittance at 550 nm in the visible region. To optimize the selectivity, the optical spectrum of this film was correlated with a theoretical model by combining the contributions of the Mie-Gans absorption-based localized surface plasmon resonance and reflections by the interfaces of the heterogeneous layers and the nanoparticles in the film. Through comparison of the composite and double layer coating method, the difference of the nanoscale distances between nanoparticles in each layer was significantly revealed. It is worth noting that the nanodistance between the nanoparticles decreased in the double layer film, which enhanced the optical properties of the film, yielding a haze value of 1% or less without any additional process. These results are very attractive for the nanocomposite coating process, which would lead to industrial fields of NIR shielding and thermo-medical applications.

**PACS:**

78.67.Sc; 78.67.Bf; 81.15.-z

## Background

Tungsten bronze nanoparticles such as tungsten trioxide doped with alkali metals have selective optical absorption properties in the near-infrared region, leading to the synthesis of various morphologies and new compounds including nanorods [1,2], nanowires [3], and nanosheets [4]. Although the optical characteristics of solutions including tungsten bronze compounds have been previously analyzed [5], additional data are essential to fully understand the absorption and reflection-induced optical characteristics for the composite coating film application.

This study has attempted to clarify the near-infrared absorption characteristics of the film using a theoretical model that considers the localized surface plasmon resonance(LSPR)-induced absorption [6], scattering [7] caused by nanoparticles, and an interlayer refractive index-induced reflection [8]. Absorption characteristics in the near-infrared region generally originate from the LSPR and can be predicted using the Mie-Gans theory [9] with the following factors proving influential: the aspect ratio [5], the electron deficiency [10,11] of the tungsten bronze compounds according to nonstoichiometric compositions, the types of doped positive-ion metals [12,13], and the purity of the tungsten bronze compounds as determined by the annealing condition [14]. Although these parameters are well defined, they focus on rather qualitative aspects confined to the material itself. The optical characteristics based on quantitative data such as the number of nanoparticles, the interference of the medium, and the internanoparticle distance must be understood.

Therefore, this study quantitatively defined these parameters based on simulated results and plotted a spectrum ranging from the visible to the near-infrared region using correlations with a theoretical model. Because simultaneously observing the selective optical transmittance in both the visible and near-infrared regions is difficult, the two regions have been analyzed using a single index, the solar transmittance selectivity. In particular, the effects of primary factors such as the internanoparticle nanodistance have been analyzed using a theoretical model-based optical spectrum.

This investigation utilized theoretically required quantitative relations and sought ways to enhance the processability. To fabricate films with a low haze, different processing conditions were tested. For these studies, a film was fabricated from nonstoichiometric cesium-doped tungsten trioxide (Cs_0.33_WO_3_) nanoparticles synthesized using a solid reaction [15] and bead milling method [16] using a composite layer coating and a novel double layer coating. Then, the optical absorption characteristics from the visible to near-infrared regions were compared to examine the effect of distance between Cs_0.33_WO_3_ nanoparticles in each layer.

## Methods

**Figure 1:**
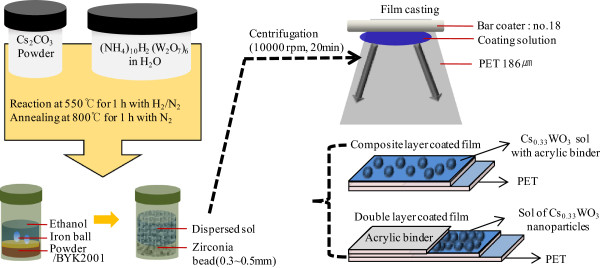
**Schematic fabrication of NIR absorption films containing Cs**_**0.33**_**WO**_**3 **_**nanoparticles.**

Figure 1 provides a schematic representation of the manufacturing process and illustrates the composition of the film layer. Ammonium tungstate ((NH_4_)_10_H_2_(W_2_O_7_)_6_, 99.99% purity) and cesium carbonate (Cs_2_CO_3_, 99.9% purity trace metal basis) were used as precursors. These materials were each dissolved in distilled water and stirred for 1 h at room temperature, and two solutions were well mixed in a ceramic crucible. This mixture was dried at 180°C for 8 h in a heating chamber (model ON-O2GW, JEIO TECH, Seoul, South Korea). The prepared powder was heated at 550°C for 1 h under a flowing H_2_/N_2_ gas mixture (H_2_/N_2_ = 90/10 cc/min) and annealed at 800°C for 1 h under a N_2_ gas flow (N_2_ = 100 cc/min) in a vacuum furnace (model DVF-1600s, DAE HEUNG SCIENCE, Incheon, South Korea). Dark blue tungsten oxide powders were obtained and analyzed via X-ray diffraction (XRD) (model x18xhf22, JEOL, Akishima, Tokyo, Japan) at 1°/min between 0° and 90°. The powder was mixed with a dispersing agent (BYK2001) in ethanol, and a turbo-mill (model 8000D, SPEX, Metuchen, NJ, USA) with an iron ball (20 mm) and zirconia bead (0.3 mm, ZrO_2_ 94.5%, Y_2_O_3_ 5.1%) was used for top-down stepwise grinding for 4 h.

The composite layer-coated film was prepared using a mixture of dispersed sol and acrylic UV-curing binder. A rotating mixer (model MS 3basic, IKA, Nara, Japan) was used, and the polyethylene terephthalate (PET, film thickness = 186 μm) substrate was coated using the bar casting method. The coated film was dried at 80°C for 1 min in a heating chamber and illuminated using UV-curing equipment (model LZ-U1O1DCH, LICHTZEN, Gyeonggi-do, South Korea) at an intensity of 800 W/cm for 20 s. To produce the double layer-coated film, dispersed Cs_0.33_WO_3_ sol was first coated on PET substrate, and the UV binder was coated using the bar casting method.

The thickness was measured using the cross-sectional length of each film via scanning electron microscopy (SEM, JSM-6700 F, JEOL). The optical properties were examined using a UV/VIS/near-infrared (NIR) spectrophotometer (model Cary 5000, Varian Australia Pty. Ltd., Mulgrave, Australia) in the range of 300 ~ 3,300 nm. The nanodistance of the internanoparticles was measured by a transmission electron microscope (TEM, JEM-2100 F, JEOL Ltd.).

## Results and discussion

**Figure 2:**
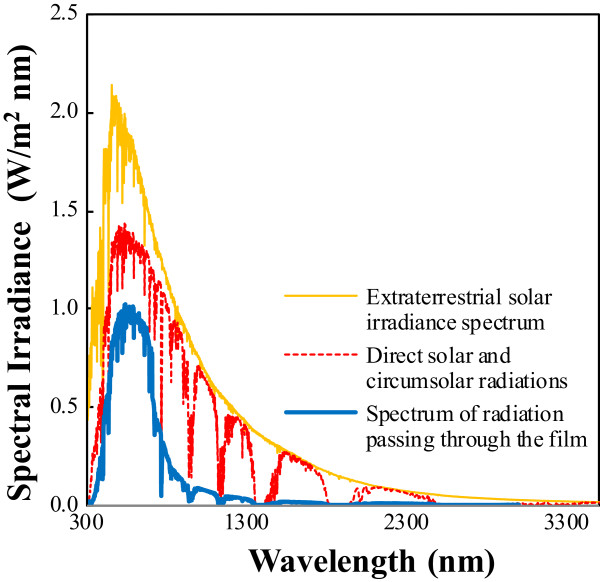
Spectral profiles from solar irradiance and that passing through the film fabricated by double layer coating method.

The solar energy spectrum in all regions was based on ASTM G173-03 as indicated in Figure 2. The solar shielding characteristics were analyzed using the solar transmittance selectivity (STS) based on the transmittance deviation (*T*_Vis_ (%), *T*_NIR_ (%)) in the visible and near-infrared regions. As indicated in Equation 1, STS refers to a factor that designates the transparent near-infrared absorption performance, while the perfect shielding film is a factor that represents the selectivity of the near-infrared absorption performance against visible rays, not the absorption performance of the solar shielding spectrum in all regions. When STS of the film is ‘1’ as an ideal film, it has 100% visible transmittance and 0% near-infrared transmittance. Therefore, this study attempted to obtain a factor that affects the performance of the film of highest selectivity with an STS approaching ‘1’.

(1) $$\mathrm{STS}=\frac{1}{2}\left(1+\frac{\int_{\mathrm{UV}}^{\mathrm{Vis}}T_{\mathrm{Vis}}\left(\%\right)-\int_{\mathrm{Vis}}^{\mathrm{NIR}}T_{\mathrm{NIR}}\left(\%\right)}{100}\right)$$

As in the brief illustration provided in Figure 1, a total light transmission and shielding (LTS) function (*T*_total_) from the visible to near-infrared regions has been proposed by summing the optical absorption and reflection-induced contribution terms using a tungsten bronze compound-based film. The contribution from the optical absorption of the film (*T*_absorption_) was determined using the Mie-Gans LSPR theory. The scattering reflection (*T*_scattering_) by the nanoparticles in the coated layer and reflection (*T*_multilayer_) based on differences of refractive index between the layers were included. The LTS function is provided in Equation 2. The factors required by various models have been quantitatively measured and are listed in Table 1.

**Table 1:** Parameters used for calculating optical shielding property of the coated film

**Thickness of the coated layer [nm]**	**Distance between nanocrystals [nm]**	**Mean diameter of nanocrystals [nm]**	**Dielectric constant of medium**	**Refractive index of the coating layer**	**Refractive index of the nanocrystals**	**Refractive index of PET substrate**
5,270	7.19	39.70	8.63	1.47	2.1	1.58

(2) $$T_{\mathrm{total}}=T_{\mathrm{absorption}}+T_{\mathrm{double}\;\mathrm{layer}}+T_{\mathrm{scattering}}$$

### Incident light absorption by the LSPR

According to the Mie-Gans theory [9,17,18], the absorption behavior of oval particles in solution is based on a dipole approximation. Thus, the absorption characteristics of *N* particles in a volume *V* against a film of a given thickness (*L*) according to the wavelength (*λ*) of incident light can be explained by Equation 3 as follows:

(3) $$T_{\mathrm{absorption}}=\frac{0.2895\;\pi \;\mathrm{NVL}\;}{\lambda }\epsilon_{m}^{3/2}\sum_{j}\frac{\left(\frac{1}{P_{j}^{2}}\right)\;\epsilon_{2}}{{\left[\epsilon_{1}+\left\{\frac{1-P_{j}}{P_{j}}\right\}\epsilon_{m}\right]}^{2}+\epsilon_{2}^{2}}$$

The thickness has been set using statistical image analysis of the measurement results obtained via SEM with image J software. In addition, *ϵ*_m_, *ϵ*_1_, and *ϵ*_2_ refer to the dielectric constant of each medium, the real number term, and the imaginary number term in the dielectric function, respectively, and can be derived as follows:

(4) $$\epsilon \left(\omega \right)=\epsilon_{1}\left(\omega \right)+i\epsilon_{2}\left(\omega \right)=1-\frac{\omega_{p}^{2}}{\omega^{2}+\mathrm{i\gamma \omega}}$$

**Figure 3:**
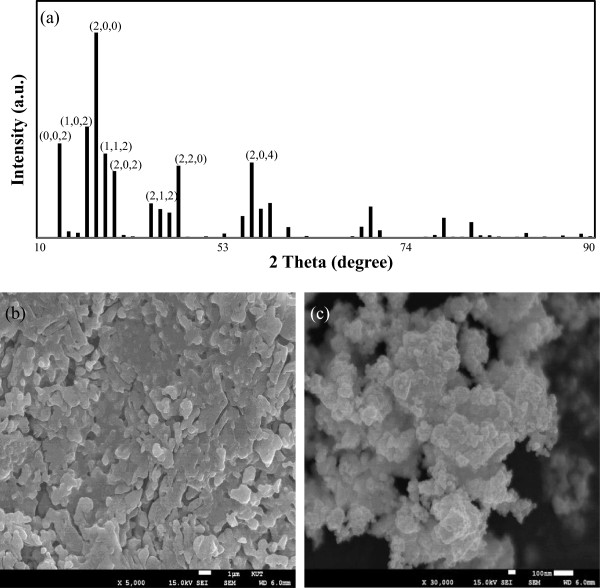
**XRD patterns and SEM images.** XRD patterns **(a)** and SEM images of as-prepared Cs_0.33_WO_3_ before **(b)** and after **(c)** the stepwise bead milling process for randomly shaped nanoparticles.

The parameters for each incident light frequency (*ω*), volume plasma frequency (*ω*_p_), and collision frequency (*γ*) are closely related to the number density (*ϱ*) and conductivity (*ζ*) of the free electrons and were computed using Equations 5 and 6 as follows:

(5) $$\omega_{p}=\sqrt{\frac{\varrho e^{2}}{\epsilon_{0}m_{e}}}$$

(6) $$\gamma =\frac{1}{\tau }=\frac{e^{2}}{\zeta m_{e}}$$

in which *τ*, *ϵ*_0_, and *m*_e_ are the scattering time for the electrons, the transmittance under vacuum conditions, and the effective electron mass, respectively. The number density of free electrons is a property intrinsic to a given material and is calculated using $\varrho =\frac{2\times 10^{21}}{\;V_{\mathrm{cell}}\;}$ in which *V*_cell_ is the unit cell volume of the Cs_0.33_WO_3_ nanoparticle. As indicated in Figure 3, the unit cell dimensions of *α* and *β* axes were 0.74 and 0.76 nm, respectively. The XRD patterns were well matched with those of a hexagonal Cs_0.33_WO_3_ nanoparticle found in related records (PDF 01-081-1244), and *V*_cell_ was used as 0.361 nm^3^[19].

The LSPR is reportedly influenced by the morphology. In tungsten oxide, however, its effect on the NIR absorption characteristics is minor [7]. To consider the randomly shaped nanoparticles fabricated through a solid reaction, depolarization factors were also used as indicated in Equation 7, which assumes an aspect ratio-related factor (*S*) of 0.417.

(7) $$P_{j}=\frac{\left(1-s^{2}\right)}{s^{2}}\left[\frac{1}{s}\ln\left(\frac{1+s}{1-s}\right)-1\right]$$

### Incident light reflection by the difference in refractive indices between the layers

The incident light passing through the coated film is interrupted due to differences in the light velocity caused by differences in the interlayer refractive index. In a double layer-coated film, this interruption occurs between the layers of different materials (the tungsten bronze-coated layer (1) and the PET substrate (2)), which partially reflect the incident light. As stated in Equation 8, the contribution for the interlayer reflection (*T*_multilayer_) has been considered.

(8) $$T_{\mathrm{double}\;\mathrm{layer}}=\frac{r_{1}^{2}+r_{2}^{2}+2r_{1}r_{2}\cos2\theta \prime }{1+r_{1}^{2}r_{2}^{2}+2r_{1}r_{2}\cos2\theta \prime }$$

in which *r*_1_ and *r*_2_ are the refractive indices of the coated layer and PET substrate, respectively, while *θ*′ refers to the phase thickness of the coated layer. The reflectance can be calculated using the refractive indices of the coated layer (*n*_1_) and PET substrate (*n*_2_) as stated in Equations 9, 10, and 11.

(9) $$r_{1}=\frac{n_{0}-n_{1}}{n_{0}+n_{1}}$$

(10) $$r_{2}=\frac{n_{1}-n_{2}}{n_{1}+n_{2}}$$

(11) $$\theta \prime =\frac{2\pi n_{1}L}{\lambda }$$

### Incident light scattering according to the size of the nanoparticles

Figure 3 reveals the mean diameter of Cs_0.33_WO_3_ nanoparticles, which was determined using the image J obtained through TEM and SEM measurements. In a top-down synthesis via the grinding method, the particle sizes are broadly distributed. In these particles, Rayleigh scattering (*T*_scattering_) occurs as indicated in Equation 12:

(12) $$T_{\mathrm{scattering}}=-\log_{10}\left[\left(\frac{1+\cos^{2}\theta }{2R^{2}}\right){\left(\frac{2\pi }{\lambda }\right)}^{4}{\left(\frac{n^{2}-1}{n^{2}+2}\right)}^{2}{\left(\frac{d}{2}\right)}^{6}\right]$$

in which *θ* is the scattering angle assumed to be 90°, while *n* and *d* are the refractive indices of the nanoparticle. The term *R* refers to the internanoparticle distance and was calculated using Equation 13 that considers the volume of nanoparticle (*V*_p_) and the residual weight (TGA (g)) as measured via thermogravimetric analysis (TGA).

(13) $$R=\frac{\mathrm{TGA}\left(g\right)\;M_{w}N_{A}}{\;V_{p}/V_{\mathrm{cell}}}$$

### The total light transmission and shielding functions for the tungsten bronze film

**Figure 4:**
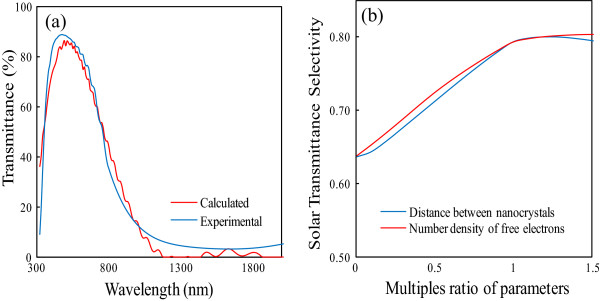
**LTS characteristics. (a)** Plots of calculated and measured spectra of Cs_0.33_WO_3_ film in the range from UV to NIR region and **(b)** effects of number density of free electrons and distance between nanoparticles in the film on solar transmittance selectivity.

The total LTS characteristics have been measured using the absorbance of the transparent near-infrared absorption film from the visible to the infrared regions. In addition, the calculated value is typically slightly below the measured value due to specimen nonuniformity and plasmon damping caused by surface electron scattering [20]. To consider this type of damping, the results were calibrated via numerical analysis. However, the hard-to-measure electrical conductivity of the nanoparticle was set at 1.03 × 10^−8^ Ω^−1^ cm^−1^. Thus, the measured values (blue line) were almost identical to the spectral results (red line) using LTS as indicated in Figure 4.

The effect of the internanoparticle distance is demonstrated in Figure 4, which shows the solar transmittance selectivity for the multiple ratios of parameters. The multiple ratio with ‘1’ of the number density of free electrons was determined from the solution-based results (i.e., *ϱ* = 6.3 × 10^21^ cm^−3^) [5]. Unfortunately, the distance of nanoparticles was not reported before; we used 8 nm as the standard parameter. As the distance between nanoparticles is too small (<1 of multiple ratio), the solar transmittance selectivity is also decreased due to the loss of transmittance in visible range. According to this sensitivity analysis, we find that the distance of nanoparticles has a pronounced effect on the solar transmittance selectivity in common with those from the number density of free electrons. Moreover, one can reasonably state that the number density on the thin layers is more important than the content of the coated layer throughout the entire volume. Therefore, this study fabricated a double layer-coated film using the facile dense layer of nanoparticles [21] and attempted to analyze the factors that quantitatively influence its optical characteristics.

### The quantitative evaluation of a novel double layer-coated film

**Figure 5:**
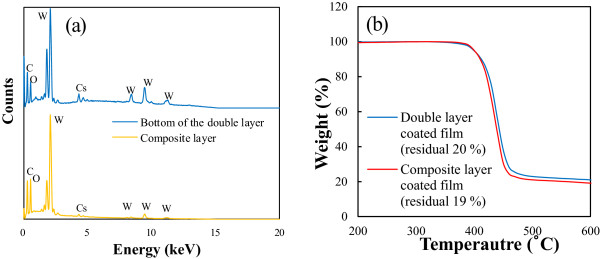
**Comparison of the composite and double layer by EDS and TGA analysis. (a)** EDS spectra and **(b)** TGA curves of the composite layer and the lower layer of the double layer-coated film.

As explained by the energy-dispersive X-ray spectroscopy (EDS) analysis of a section of the coated layer depicted in Figure 5, the contents of tungsten compound in the coating layer of the double layer-coated film exceed those in the composite layer. Despite measurement errors (1%), reproducible results can be obtained as stated in Table 2, which indicates that the nanoparticles in the double-coated layers are in close proximity. The residual nanoparticle content was determined via the TGA measurement and confirmed that the content of the composite layer-coated materials was almost identical to that of the double layer-coated nanoparticles (<1%). This result indicates that the nanoparticles in the double layer are more densely distributed than those in the composite layer, and the number density of the particles in the horizontal layer, not the number on the coated layer, is larger.

**Table 2:** EDS results of the coated layer in the composite layer and double layer films

	**Double layer-coated film**	**Composite layer-coated film**
	**[weight %]**	**[weight %]**
Carbon K shell	41.50	42.68
Oxygen K shell	23.77	38.81
Cesium L shell	10.32	2.94
Tungsten M shell	24.41	15.57
Total	100.00	100

**Figure 6:**
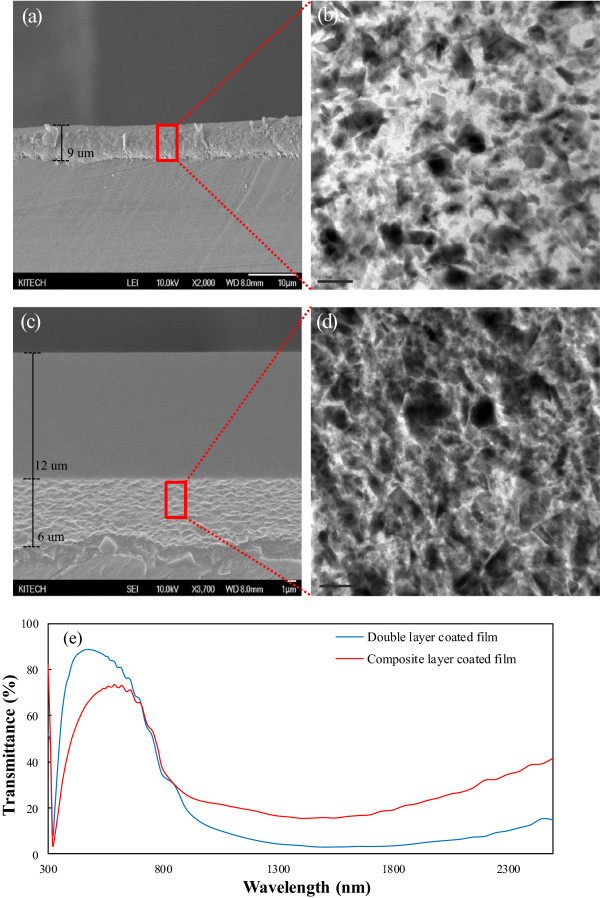
**Cross-sectional images and spectra of the Cs**_**0.33**_**WO**_**3**_-**coated films.** The cross-sectional SEM and TEM images of the Cs_0.33_WO_3_**-**coated film fabricated by composite layer **(a, b)** and double layer coating method **(c, d)** and spectra of the films fabricated by different methods from UV to NIR region **(e)**, respectively.

In addition, Figure 6 confirms the randomly shaped nanoparticles via SEM measurements, and the thickness of the coated layer, clearly cut according to the SEM and TEM cross-sectioned diagram, was measured. The thickness of the coated layer is related to the total volume of the layer of Cs_0.33_WO_3_ nanoparticles. Particularly, the spectra of the two different films have a significant deviation in the range of UV to NIR region, which implies that the number density of the nanoparticles in the double layer is larger than that of composite-coated layer in the same number.

Moreover, the haze was measured using the drying conditions of each film as stated in Table 3 to analyze the processability of the coated film. High haze was observed in the composite layer-coated film under typical thermal drying conditions. While the haze value of coating film depends on somewhat subjective conditions, such as the surface roughness and type and composition ratio of the dispersants in the coated materials [22], however, low haze could be detected using thermal drying under vacuum. Meanwhile, in a double layer-coated film constructed from layers containing individual materials, the lowest haze of the film was observed compared to that from the composite layer coating due to the absence of surface roughness by nanoparticles in the surface as shown in SEM cross-sectioned images. Thus, from the perspective of haze value, the double layer-coated film is less sensitive to the effect of surface roughness.

**Table 3:** Haze values by varying the drying conditions and different coating methods

	**Double layer-coated film dried at 80°C**	**Composite layer-coated film dried at**			
		**80°C**	**90°C**	**100°C**	**100°C (vacuum oven)**
Haze value	<1.00	7.28	5.28	3.76	1.07

## Conclusions

Using a LTS model based on the Mie-Gans theory, double layer reflection, and Rayleigh scattering, this study quantitatively analyzed the contributions for high near-infrared absorption film with high transparency. After determining the effects of internanoparticle distance within the layer on the STS, a novel double layer-coated film was fabricated with a small nanodistance between Cs_0.33_WO_3_ tungsten bronze nanoparticles. Considering the total solar energy spectrum, 380 W/m^2^ of solar absorption energy was estimated. Moreover, the double layer-coated film has 80% visible transmittance at 550 nm, 10% near-infrared transmittance at 1,000 nm, and low haze with 1% or less. In addition, the STS of the film was 0.793, and thus, the double layer-coated film was found to have excellent near-infrared absorption compared with that of a composite layer-coated film (0.696).

## Competing interests

The authors declare that they have no competing interests.

## Authors’ contributions

SYL performed the theoretical calculations and overall experiment. The nanoparticles were prepared by JYK, and HJS optimized their physical properties. JYL participated in drafting the manuscript and technical support. SL participated in the design of experiments. KHC participated in the analysis of the optical results. Drafting of the manuscript was carried out by GS. All authors read and approved the final manuscript.
